# Supplementary material for: Community structure-regulation coupling reveals optimal information diffusion
Source: Nat Commun. 2026 Jun 2;17:4879. doi: 10.1038/s41467-026-73665-1 (PMC13230782; doi:10.1038/s41467-026-73665-1)
Supplement: Supplementary file 1 — Supplementary Information [file 41467_2026_73665_MOESM1_ESM.pdf]

# Supplemental Material: "Community structure–regulation coupling reveals optimal information diffusion"

Xiaojie Chen,[1,2,*] Meiling Xie,[1,*] Jun Meng,[3] Sheng Fang,[1] Xiaosong Chen,[1,4] Jürgen Kurths,[5,6] Jan Nagler,[7,†] and Jingfang Fan[1,5,8,‡]

[1]*School of Systems Science/Institute of Nonequilibrium Systems, Beijing Normal University, 100875 Beijing, China*

[2]*School of Physics, Hubei University, 430062, Wuhan, Hubei, China*

[3]*State Key Laboratory of Earth System Numerical Modeling and Application, Institute of Atmospheric Physics, Chinese Academy of Sciences, Beijing, 100029, China.*

[4]*Institute for Advanced Study in Physics, Zhejiang University, Hangzhou 310058, China*

[5]*Potsdam Institute for Climate Impact Research, 14412 Potsdam, Germany*

[6]*Department of Physics, Humboldt University, 10099 Berlin, Germany*

[7]*Deep Dynamics, Centre for Human and Machine Intelligence, Frankfurt School of Finance & Management, 60322 Frankfurt am Main, Germany*

[8]*State Key Laboratory of Marine Environmental Science, Xiamen University, 361005 Xiamen, China*

(Dated: May 14, 2026)

## I. SIS MODEL FOR INFORMATION PROPAGATION

The Susceptible–Infected–Susceptible (SIS) model for information propagation assumes that individuals do not retain information permanently, but can alternate between uninformed and informed states through the cycle $S \to I \to S$. This cyclical process captures the realistic phenomenon of information decay and re-acquisition, where awareness may fade over time and later be renewed by social exposure. The fundamental transitions are,

$$\begin{aligned} S + I &\xrightarrow{\beta} 2I, \\ I &\xrightarrow{\chi} S, \end{aligned} \tag{S1}$$

where $\beta$ denotes the information transmission rate—how rapidly uninformed individuals acquire information through contact with informed peers—and $\chi$ represents the forgetting rate, quantifying how quickly informed individuals lose or disregard the information. For sufficiently large $\beta$ or small $\chi$, the information can persist indefinitely within the population.

Applying the law of mass action, the deterministic mean-field equations of the SIS information propagation model read,

$$\frac{d\rho^S}{dt} = -\beta\rho^S\rho^I + \chi\rho^I, \tag{S2}$$

where $\rho^S$ and $\rho^I$ are the densities of susceptible and informed individuals, respectively, satisfying the normalization $\rho^S + \rho^I = 1$. The fraction of informed individuals evolves according to $\frac{d\rho^I}{dt} = \beta\rho^S\rho^I - \chi\rho^I$.

**Degree-based mean-field (DBMF) formulation.** In social systems, the heterogeneous connectivity structure significantly influences how information spreads. Within the DBMF approach, the SIS dynamics are described by the probability $\rho_k^I(t)$ that a node of degree $k$ is informed at time $t$. Following [1], the dynamical equation becomes,

$$\frac{d\rho_k^I(t)}{dt} = -\rho_k^I(t) + \lambda k \left[1 - \rho_k^I(t)\right] \sum_{k'} P(k'|k)\rho_{k'}^I(t), \tag{S3}$$

where time is rescaled by $\chi^{-1}$ so that the forgetting rate is unity, and the effective spreading rate $\lambda = \beta/\chi$ measures the balance between information acquisition and loss. A critical $\lambda_c$ separates regimes of vanishing versus sustained information presence in the system.

* These two authors contributed equally

† jan.nagler@gmail.com

‡ jingfang@bnu.edu.cn

**Two-modular network extension.** To investigate the effects of community structure, we extend the SIS framework to a two-modular network characterized by mixing parameter $\mu$, representing two social groups or information domains, denoted $A$ and $B$. The informed (uninformed) densities in each community are $\rho^{I(S),A(B)}$. The corresponding DBMF equations are

$$\frac{d\rho^{I,A(B)}_{k_{\text{intra}}k_{\text{inter}}}(t)}{dt} = -\,\rho^{I,A(B)}_{k_{\text{intra}}k_{\text{inter}}}(t) + \lambda\big(1-\rho^{I,A(B)}_{k_{\text{intra}}k_{\text{inter}}}(t)\big) \times \left[\omega_{\text{intra}}k_{\text{intra}}\sum_{k'=1}^{k_{\text{intra}}}\frac{P_{\text{intra}}(k')k'}{z_{\text{intra}}}\rho^{I,A(B)}_{k':}(t) + \omega_{\text{inter}}k_{\text{inter}}\sum_{k'=1}^{k_{\text{inter}}}\frac{P_{\text{inter}}(k')k'}{z_{\text{inter}}}\rho^{I,B(A)}_{:k'}(t)\right], \tag{S4}$$

where $\rho^{I,A(B)}_{k':}(t) = \sum_{k''} P_{\text{inter}}(k'')\rho^{I,A(B)}_{k'k''}(t)$ and $\rho^{I,B(A)}_{:k'}(t) = \sum_{k''} P_{\text{intra}}(k'')\rho^{I,B(A)}_{k''k'}(t)$.

## II. DEGREE-BASED MEAN-FIELD APPROXIMATION FOR MULTI-MODULE NETWORKS

The Mean-Field Approximation (MFA) assumes a well-mixed connectivity pattern where local correlations and backtracking effects are ignored. Specifically, it assumes that the probability of a neighbor being active is given by the global average adoption density $\rho$ of its community, rather than the cavity probability.

For a network composed of $n$ modules, we denote the final adoption density in the $I$-th community as $\rho^I_\infty$. Following the law of mass action and incorporating degree heterogeneity, the steady-state adoption density for community $I$ is given by:

$$\rho^I_\infty = \rho^I_0 + (1-\rho^I_0)\sum_{\mathbf{k}} P(\mathbf{k}) \times \Omega_{\text{MFA}}(\mathbf{k}, \boldsymbol{\rho}_\infty), \tag{S5}$$

where $\rho^I_0$ is the initial adoption density in community $I$, and $P(\mathbf{k}) = \prod_j P_j(k_j)$ is the joint degree distribution. The term $\Omega_{\text{MFA}}$ represents the probability that a node with degree vector $\mathbf{k} = (k_1, k_2, \ldots, k_n)$ receives sufficient stimulus to adopt the information:

$$\Omega_{\text{MFA}}(\mathbf{k}, \boldsymbol{\rho}_\infty) = \sum_{\mathbf{m}\le\mathbf{k}}\left[\prod_{j=1}^{n}\mathcal{B}\left(m_j,\, k_j,\, \rho^j_\infty\right)\right]\mathcal{R}\left(\mathbf{m}, \omega, \theta, \mathbf{k}\right). \tag{S6}$$

Here, $\mathcal{B}(m_j, k_j, \rho^j_\infty) = \binom{k_j}{m_j}(\rho^j_\infty)^{m_j}(1-\rho^j_\infty)^{k_j-m_j}$ is the binomial probability that $m_j$ out of $k_j$ neighbors in community $j$ are adopted. The response function $\mathcal{R}$ determines the activation based on the total weighted stimulus $\sum_j \omega_{Ij}m_j$ exceeding the threshold.

**Special Case: Two-Module System.** For the two-module case ($n = 2$, communities $A$ and $B$), the steady-state density for community $A$ simplifies to:

$$\begin{aligned}\rho_\infty^A =& \rho_0^A + (1-\rho_0^A) \sum_{k_{\text{intra}}, k_{\text{inter}}}^{\infty} P_{\text{intra}}(k_{\text{intra}}) P_{\text{inter}}(k_{\text{inter}}) \\ &\times \sum_{m_{\text{intra}}=0}^{k_{\text{intra}}} \sum_{m_{\text{inter}}=0}^{k_{\text{inter}}} \mathcal{B}(m_{\text{intra}}, k_{\text{intra}}, \rho_\infty^A) \mathcal{B}(m_{\text{inter}}, k_{\text{inter}}, \rho_\infty^B) \mathcal{R}(\boldsymbol{m}, \boldsymbol{\omega}, \theta, k_{\text{intra}} + k_{\text{inter}}),\end{aligned} \tag{S7}$$

where $k_{\text{intra}}$ and $k_{\text{inter}}$ denote the number of links to nodes within the same community and the opposite community, respectively. A symmetric expression holds for $\rho_\infty^B$. This formulation, while computationally efficient, tends to underestimate critical thresholds in sparse networks as it neglects the cavity effect.

## III. TREE-LIKE APPROXIMATION FOR THREE-MODULE NETWORKS

To validate the generality of the community structure–regulation coupling beyond two modules, we derive the explicit Tree-Like (TL) approximation equations for a system of three interacting modules, labeled $A$, $B$, and $C$. Within the generalized $n$-module TL framework presented in the main text, the cavity probability $y_l^{(I)}$ in the $I$-th community evolves via an iterative map that accounts for all possible source communities of incoming edges. For the three-module case, the evolution equation for the cavity probability $y^{(A)}$ in module $A$ takes the explicit form:

$$\begin{aligned}y_{l+1}^{(A)} =& \rho_0^{(A)} + (1-\rho_0^{(A)}) \sum_{k_{AA}, k_{AB}, k_{AC}} \frac{k_{\text{tot}}}{z} P^{(A)}(\mathbf{k}) \\ &\times \Bigg\{ \frac{k_{AA}}{k_{\text{tot}}} \sum_{\mathbf{m}} \mathcal{B}(m_{AA}, k_{AA}-1, y_l^{(A)}) \mathcal{B}(m_{AB}, k_{AB}, y_l^{(B)}) \mathcal{B}(m_{AC}, k_{AC}, y_l^{(C)}) \mathcal{R}(\mathbf{m}, k_{\text{tot}}-1) \\ &+ \frac{k_{AB}}{k_{\text{tot}}} \sum_{\mathbf{m}} \mathcal{B}(m_{AA}, k_{AA}, y_l^{(A)}) \mathcal{B}(m_{AB}, k_{AB}-1, y_l^{(B)}) \mathcal{B}(m_{AC}, k_{AC}, y_l^{(C)}) \mathcal{R}(\mathbf{m}, k_{\text{tot}}-1) \\ &+ \frac{k_{AC}}{k_{\text{tot}}} \sum_{\mathbf{m}} \mathcal{B}(m_{AA}, k_{AA}, y_l^{(A)}) \mathcal{B}(m_{AB}, k_{AB}, y_l^{(B)}) \mathcal{B}(m_{AC}, k_{AC}-1, y_l^{(C)}) \mathcal{R}(\mathbf{m}, k_{\text{tot}}-1) \Bigg\},\end{aligned} \tag{S8}$$

where $k_{\text{tot}} = k_{AA} + k_{AB} + k_{AC}$ is the total degree of a node in module $A$, and $P^{(A)}(\mathbf{k})$ denotes the joint degree distribution. The three terms inside the braces correspond to the contributions from

edges originating within module $A$, from module $B$, and from module $C$, respectively; in each case the cavity correction subtracts one from the degree of the source community.

After iterating to the fixed point $\mathbf{y}_\infty = (y_\infty^{(A)}, y_\infty^{(B)}, y_\infty^{(C)})$, the final density of adopted nodes in module $A$ is computed as:

$$\begin{aligned}\rho_\infty^{(A)} = \rho_0^{(A)} + (1-\rho_0^{(A)}) &\sum_{k_{AA},k_{AB},k_{AC}} P^{(A)}(k_{AA},k_{AB},k_{AC}) \\ &\times \sum_{m_{AA},m_{AB},m_{AC}} \prod_{j\in\{A,B,C\}} \mathcal{B}\big(m_{Aj},\, k_{Aj},\, y_\infty^{(j)}\big) \\ &\times \mathcal{R}\big(m_{AA},\, m_{AB},\, m_{AC},\, k_{\text{tot}}\big).\end{aligned} \tag{S9}$$

The densities $\rho_\infty^{(B)}$ and $\rho_\infty^{(C)}$ for modules $B$ and $C$ are computed analogously with appropriate index permutations. As shown in fig. **15**, the theoretical predictions from the TL approximation show excellent agreement with Monte Carlo simulations across a range of mixing parameter, confirming that the community structure–regulation coupling identified in the two-module case extends to multi-module topologies.


[1] Romualdo Pastor-Satorras, Castellano, C., Van Mieghem, P. & Vespignani, A. Epidemic processes in complex networks. *Reviews of Modern Physics* **87**, 925–979 (2015).

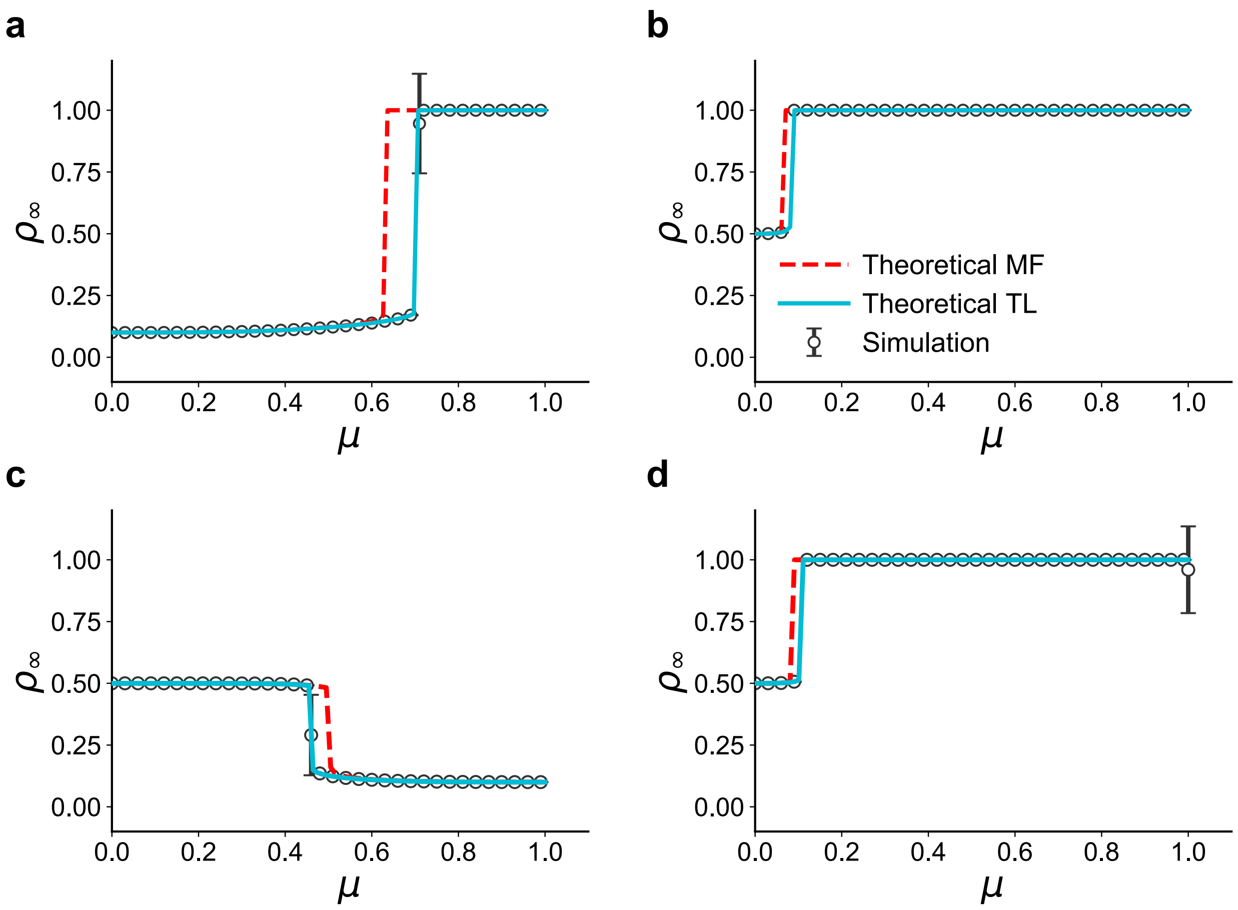


**Supplemental Fig. 1**. **Phase transition cross-sections under varying $\mu$.** Analogous to Fig. 2, panels (**a,b**) show cross sections of the phase diagram for fixed $\omega_{\text{intra}} = 0.2$ and $0.8$, respectively, while panels (**c,d**) display results for fixed $\omega_{\text{inter}} = 0.2$ and $0.8$. Results from the TL approximation (blue) agree closely with simulations (black circles with error bars indicating standard deviations), whereas MF predictions (red) overestimate the extent of the diffusion phase. Parameters: $z = 15$, $\rho_0 = 0.1$, $\theta = 0.25$, and $N = 200,000$ (simulations).

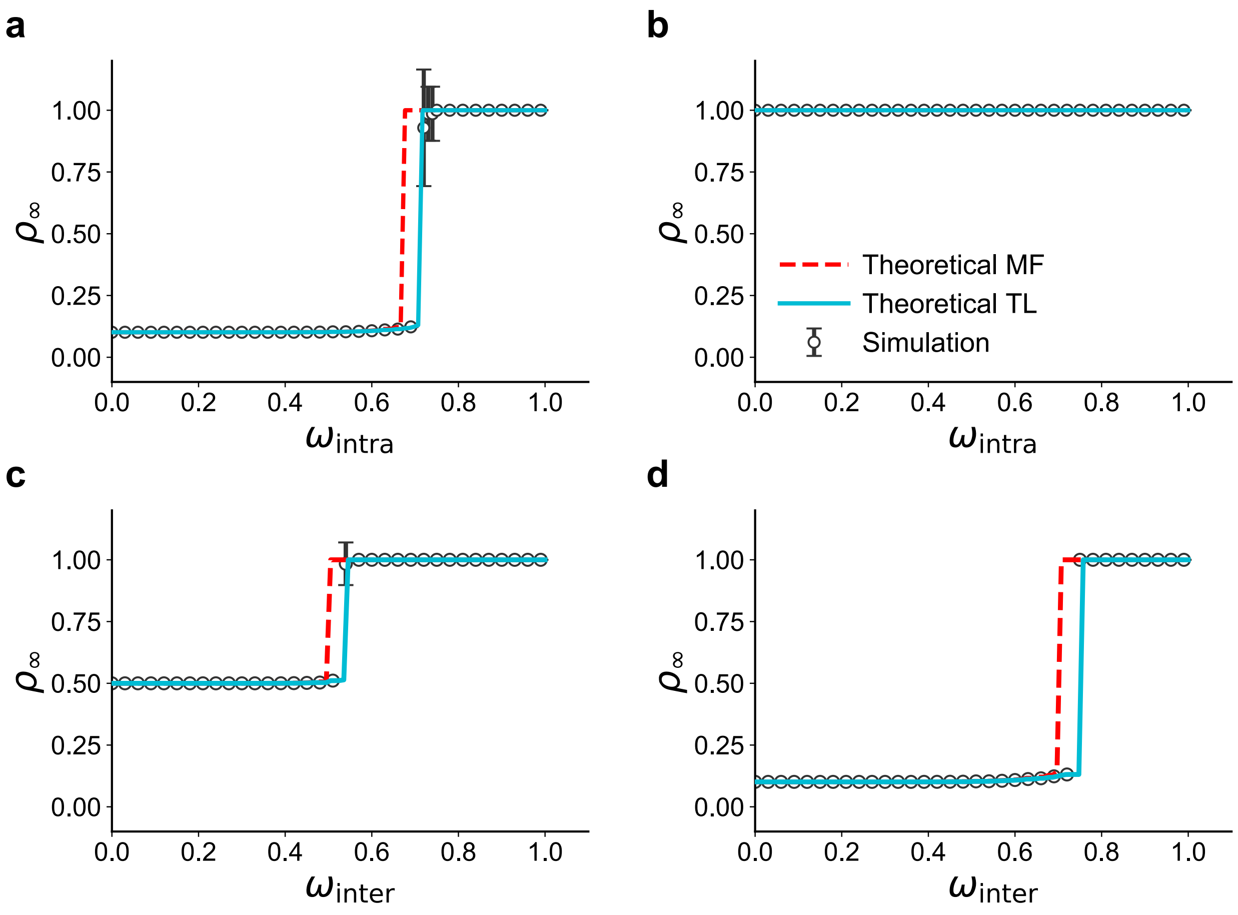


**Supplemental Fig. 2**. **Phase transition cross-sections under varying $\omega$.** Analogous to Fig. 2, panels (**a,b**) show cross sections of the phase diagram for $\mu = 0.2$ and $0.8$ with $\omega_{\text{inter}} = 1$, respectively, while panels (**c,d**) correspond to $\mu = 0.2$ and $0.8$ with $\omega_{\text{intra}} = 1$. The results illustrate how community structure influences the onset of diffusion across intra- and inter-community regulation regimes. Parameters: $z = 15$, $\rho_0 = 0.1$, $\theta = 0.25$, and $N = 200,000$ (simulations).

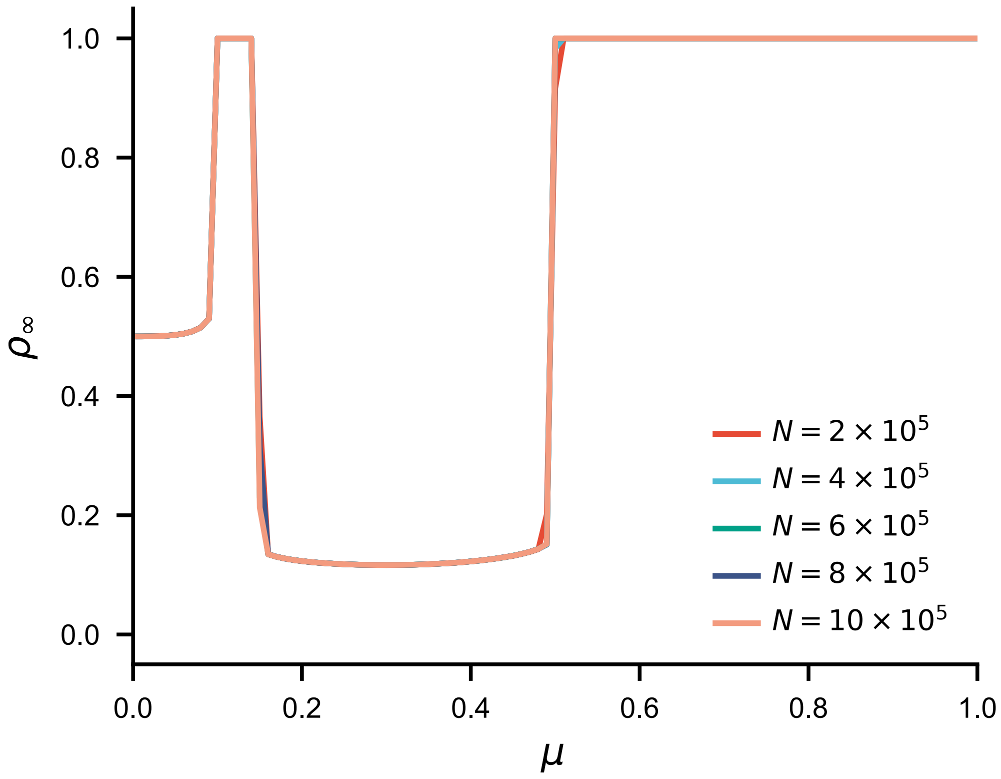


**Supplemental Fig. 3**. **Finite-size effects on diffusion dynamics.** Final adoption density $\rho_\infty$ as a function of network mixing parameter $\mu$, obtained from simulations with different system sizes $N = 2 \times 10^5$, $4 \times 10^5$, $6 \times 10^5$, $8 \times 10^5$, and $10^6$. The results show that finite-size variations have negligible influence on the phase structure. Parameters: $z = 15$, $\rho_0 = 0.1$, $\omega_{\text{intra}} = 0.7$, $\omega_{\text{inter}} = 1$, and $\theta = 0.25$.

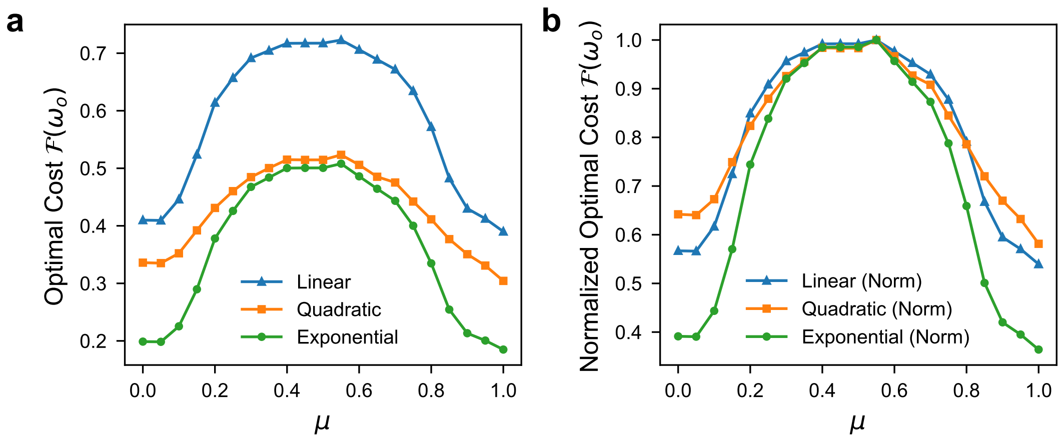


**Supplemental Fig. 4**. **Sensitivity analysis of the optimal cost $\mathcal{F}(\omega_o)$ under three different cost functions—Linear (blue triangles), Quadratic (orange squares), and Exponential (green circles)—as a function of the mixing parameter $\mu$ (by TL approximation on ER-ER-ER network).** (**a**) Optimal cost: the absolute magnitudes differ across functional forms, but all three curves exhibit a consistent non-monotonic profile with a single maximum at intermediate $\mu$. (**b**) Normalized optimal cost: after rescaling each curve to its own maximum value, the three profiles collapse onto a similar shape, confirming that the peak region consistently occurs at intermediate $\mu$. While the detailed curvature reflects the different sensitivities of the cost functions to the control parameters, the qualitative conclusion remains unchanged: the existence of an optimal intervention regime at intermediate community structure is a robust structural feature and does not depend on the specific form of the cost function. Parameters: $\theta = 0.1$, $\rho_0 = 0.17$, $z = 20$, and $N = 200,000$ (simulation).

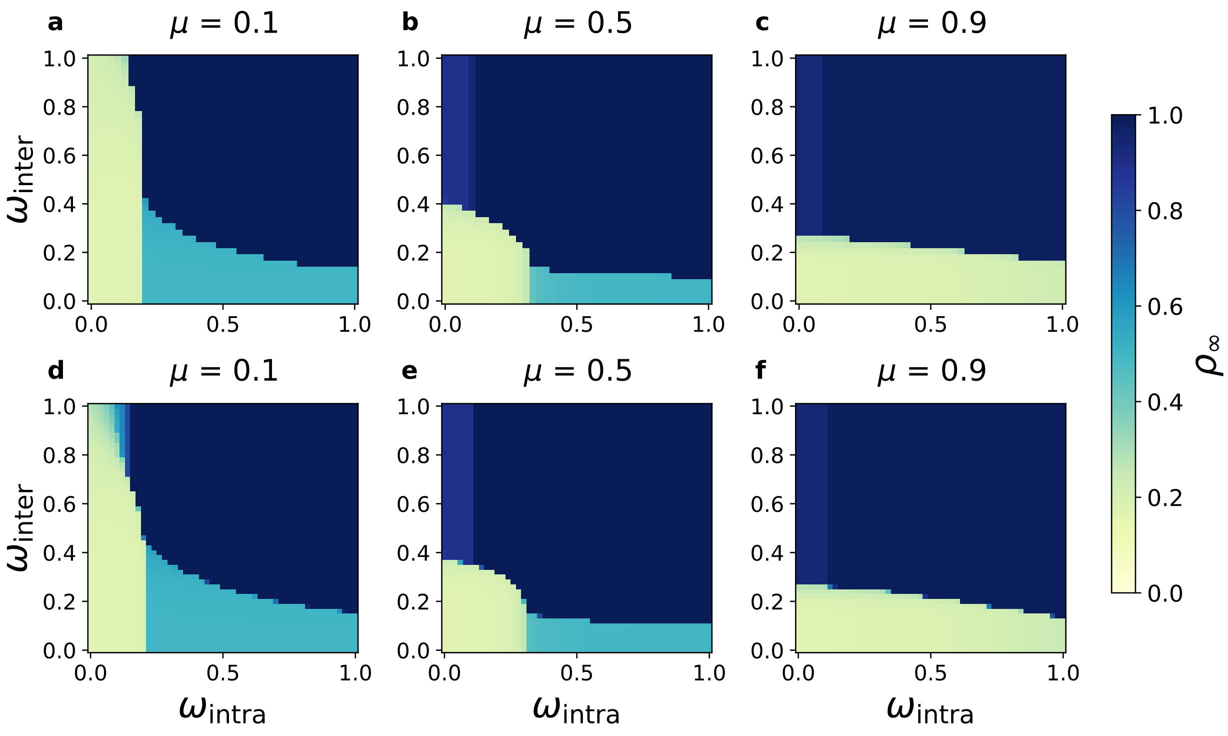


**Supplemental Fig. 5. Optimal controllability in heterogeneous modular networks.** Phase diagrams of the final adoption density $\rho_\infty$ from the TL approximation are shown in panels (**a–c**), with corresponding simulation results in (**d–f**) for modularities $\mu = 0.1$, $0.5$, and $0.9$. The intra-community degree distribution follows a Poisson law, $P_{\text{intra}}(k_{\text{intra}}) = \frac{z_{\text{intra}}^{k_{\text{intra}}} e^{-z_{\text{intra}}}}{k_{\text{intra}}!}$, while the inter-community degree distribution follows a power law, $P_{\text{inter}}(k_{\text{inter}}) \sim k_{\text{inter}}^{-\lambda_{\text{inter}}}$. The close agreement between theory and simulation demonstrates that the optimal controllability domain persists under structural heterogeneity, confirming the robustness of the MOCOF beyond homogeneous (ER–ER–ER) topologies. Parameters: $\theta = 0.1$, $\rho_0 = 0.17$, $\lambda_{\text{inter}} = 3$, $z = 20$, and $N = 200,0000$ (simulations).

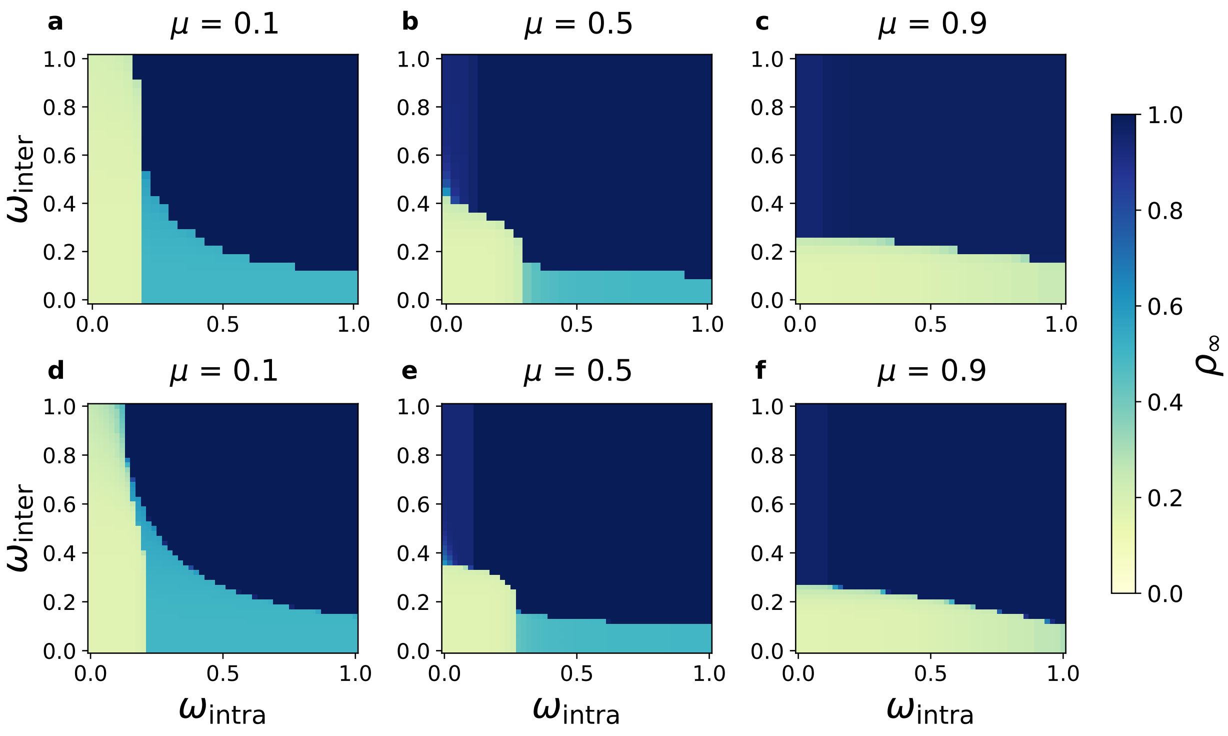


**Supplemental Fig. 6**. **Optimal intervention in ER–SF–ER networks with varying inter-community scaling.** Same setup as Supplemental Fig. 5, but with a different inter-community degree exponent, $\lambda_{\text{inter}} = 2.5$. Phase diagrams obtained via the TL approximation are shown in panels (**a–c**), and the corresponding simulation results in (**d–f**), for mixing parameters $\mu = 0.1$, $0.5$, and $0.9$, respectively. The intra-community degree distribution follows a Poisson law, $P_{\text{intra}}(k_{\text{intra}}) = \frac{z_{\text{intra}}^{k_{\text{intra}}} e^{-z_{\text{intra}}}}{k_{\text{intra}}!}$, while the inter-community degree distribution follows a power law, $P_{\text{inter}}(k_{\text{inter}}) \sim k_{\text{inter}}^{-\lambda_{\text{inter}}}$. Results confirm that the community structure–regulation coupling and optimal intervention domains remain robust under changes in the inter-community scaling exponent. Parameters: $\theta = 0.1$, $\rho_0 = 0.17$, $z = 20$, and $N = 200,000$ (simulations).

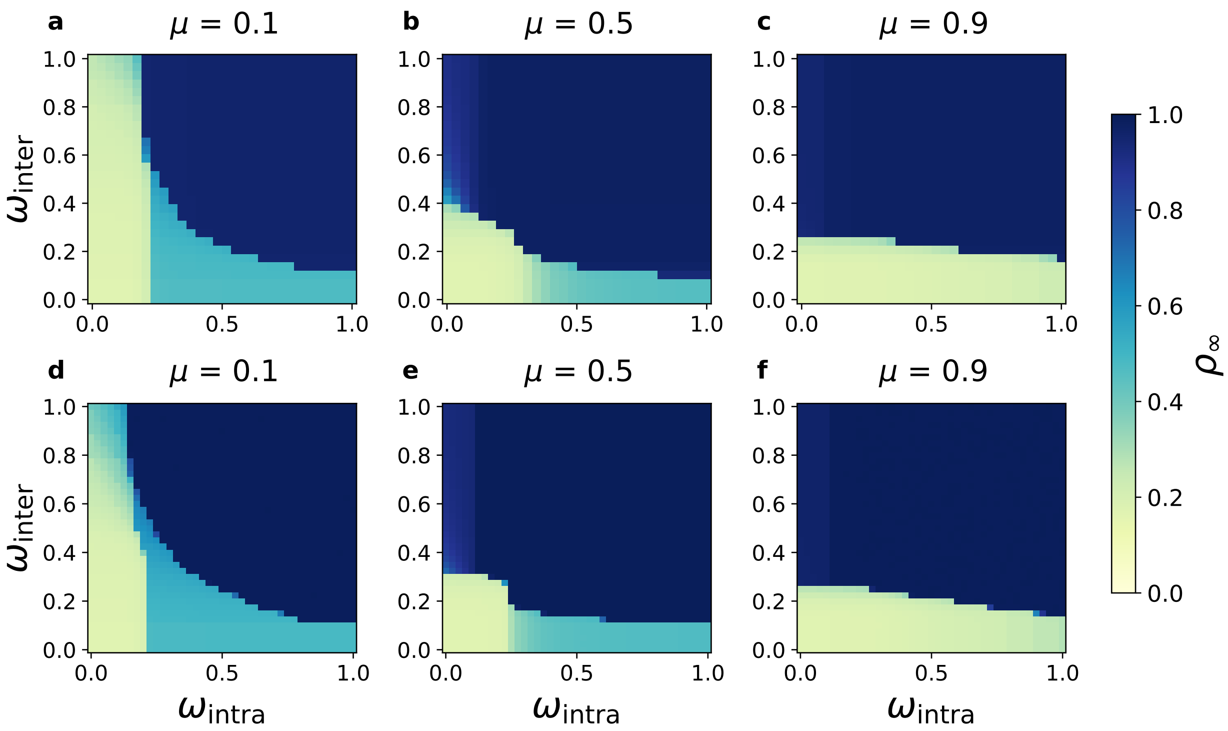


**Supplemental Fig. 7**. **Optimal intervention in SF–SF–SF networks.** Same setup as Fig. 4 but with different power-law exponents, $\lambda_{\text{intra}} = 2.5$ and $\lambda_{\text{inter}} = 2.5$. Both TL approximations (**a–c**) and simulations (**d–f**) exhibit consistent phase structures across mixing parameter levels $\mu = 0.1$, $0.5$, and $0.9$, confirming the robustness of the community structure–regulation coupling under varying network heterogeneity. Parameters: $\theta = 0.1$, $\rho_0 = 0.17$, $z = 20$, and $N = 200,000$ (simulations).

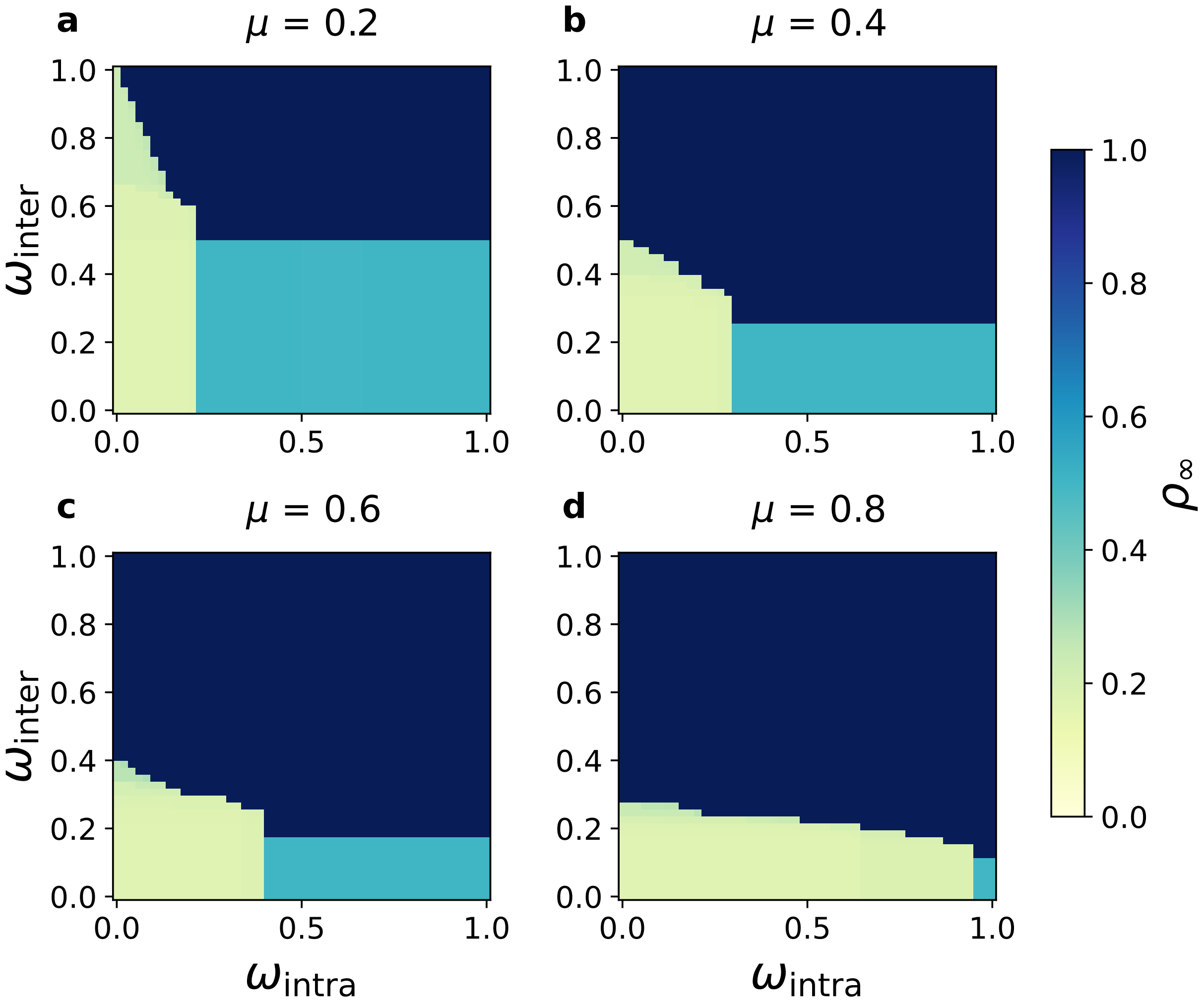


**Supplemental Fig. 8**. **Optimal intervention in RR-RR-RR networks.** Each node has a fixed degree $z = 20$, ensuring homogeneous connectivity. Phase diagrams obtained from the TL approximation are shown in panels (**a–d**) for mixing parameters $\mu = 0.2$, $0.4$, $0.6$, and $0.8$, respectively. The consistent phase boundaries across different $\mu$ values confirm that regulation is preserved in homogeneous topologies. Parameters: $\theta = 0.1$, $\rho_0 = 0.17$.

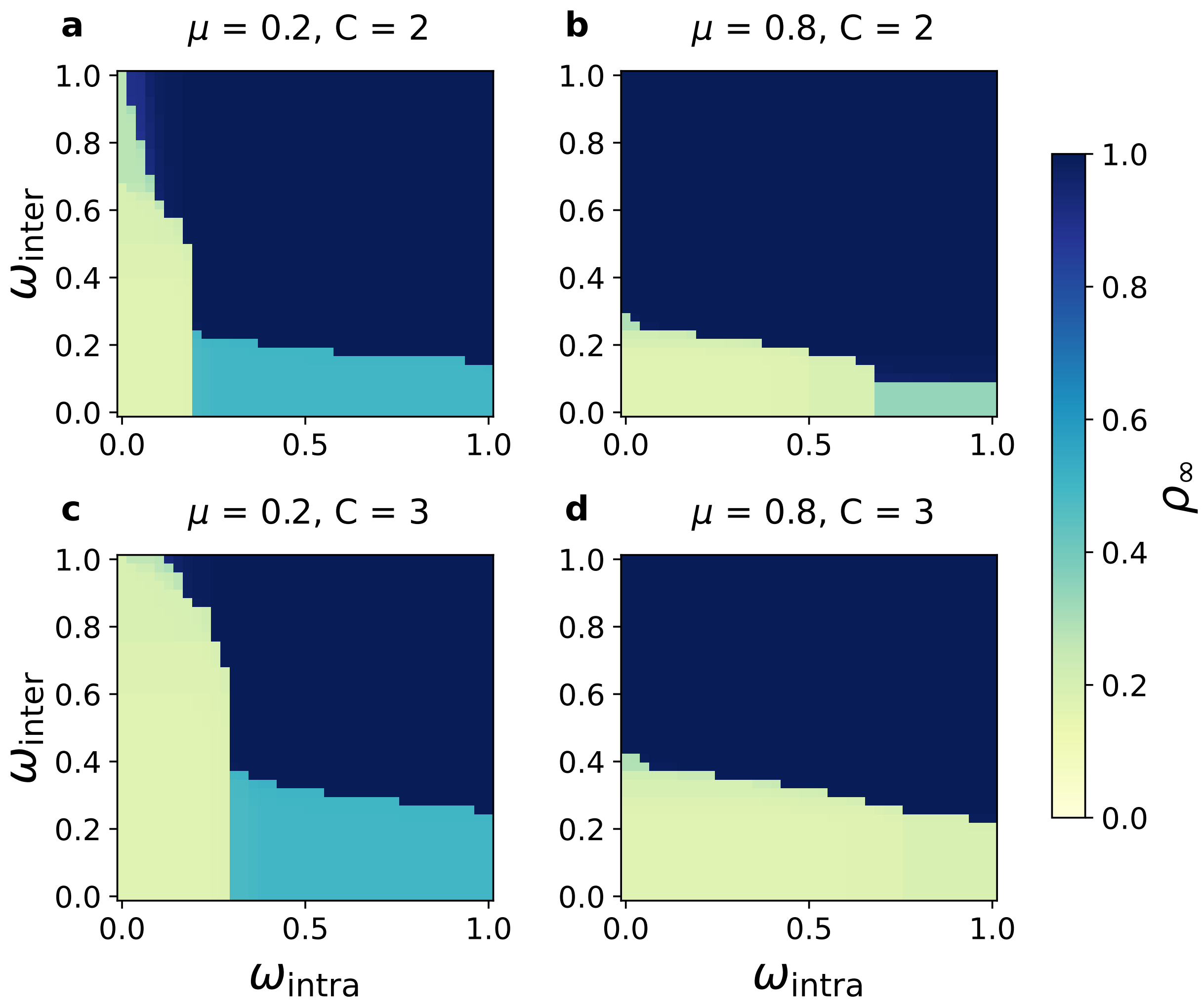


**Supplemental Fig. 9**. **Optimal intervention in ER–ER–ER networks with a fixed-threshold rule.** Under the fixed-number threshold rule, a susceptible node becomes adopted once the number of its adopted neighbors exceeds a constant threshold $C$. The response function is defined as $\mathcal{R}(\boldsymbol{m}, \boldsymbol{\omega}, C) = \begin{cases} 1, & \boldsymbol{m} \cdot \boldsymbol{\omega} > C, \\ 0, & \text{otherwise.} \end{cases}$ Phase diagrams obtained via the TL approximation are shown for different mixing parameters $\mu$ and threshold values $C$: (**a**) $\mu = 0.2$, $C = 2$; (**b**) $\mu = 0.8$, $C = 2$; (**c**) $\mu = 0.2$, $C = 3$; (**d**) $\mu = 0.8$, $C = 3$. Parameters: $\rho_0 = 0.17$ and $z = 20$.

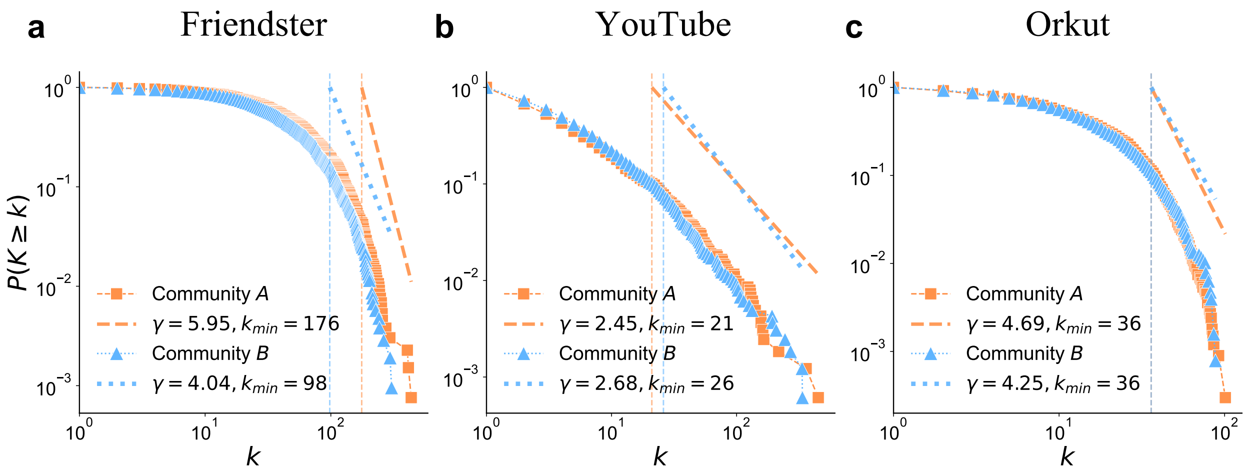


**Supplemental Fig. 10**. **Degree distributions of the two communities (Community $A$ and Community $B$) in three real-world networks.** Log–log plots of the complementary cumulative degree distributions for Community $A$ (orange) and Community $B$ (blue) in the Friendster, YouTube, and Orkut social networks. Dotted lines indicate the best-fit power-law models obtained via maximum likelihood estimation for $k \geq k_{\min}$. The analysis shows that the degree distributions of both communities deviate from strict scale-free behavior, which helps explain why transmission control in these real networks does not display sharp phase transitions.

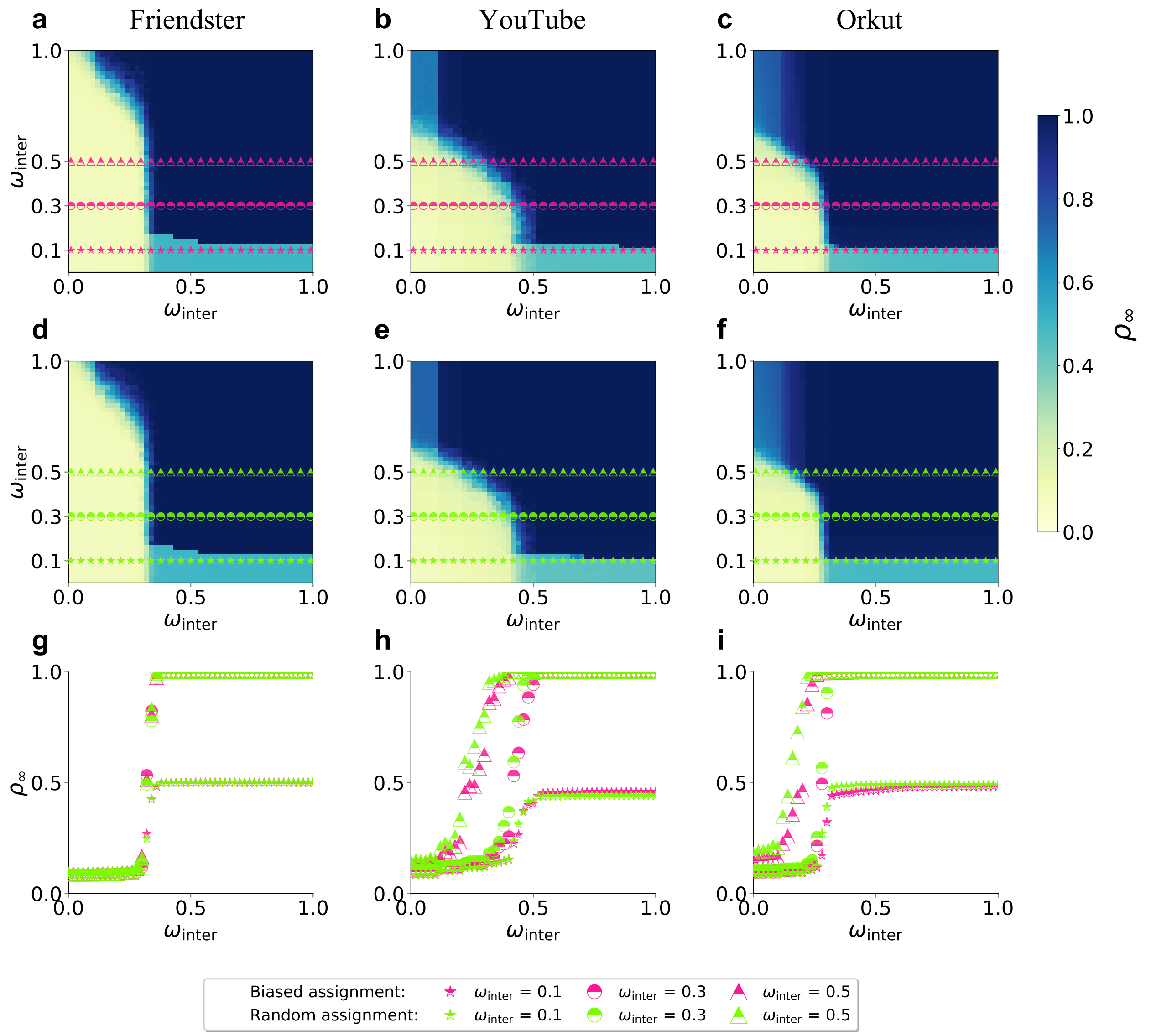


**Supplemental Fig. 11**. **Comparison of community assignment methods for overlapping nodes.** Two assignment strategies are compared: (**1**) biased assignment, where overlapping nodes are preferentially assigned to the smaller community, and (**2**) random assignment between communities. Panels (**a–c**) show phase diagrams obtained using the biased assignment method, while panels (**d–f**) display results from random assignment. Panels (**g–i**) present cross-sections for fixed $\omega_{\text{inter}}$ values (0.1, 0.3, and 0.5); pink dots correspond to the biased assignment, and green dots to random assignment. The close similarity between the two methods indicates that the influence of overlapping-node assignment on the diffusion dynamics is minor, confirming the robustness of the COSREF.

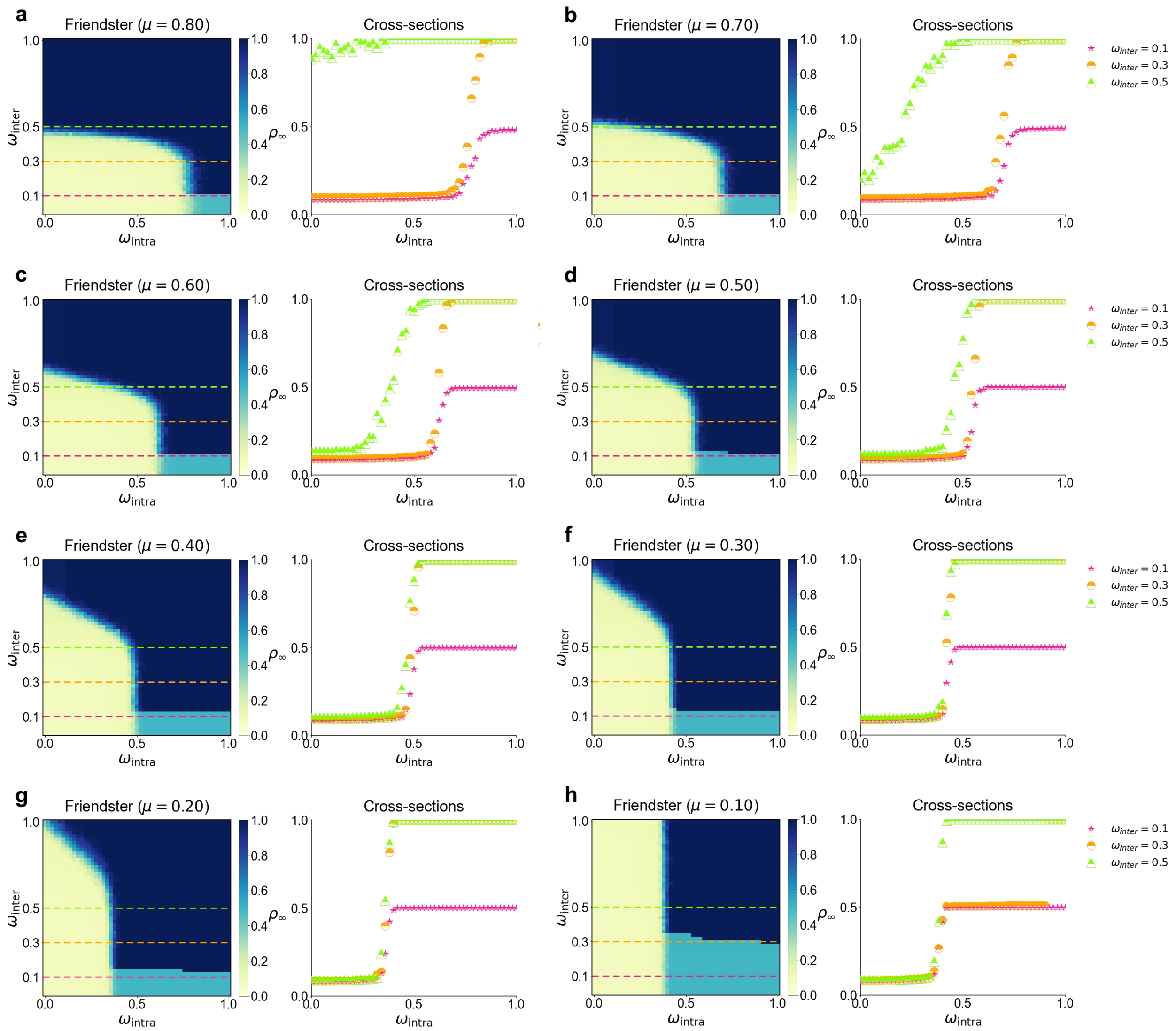
a
Friendster ($\mu = 0.80$)
Cross-sections
b
Friendster ($\mu = 0.70$)
Cross-sections
c
Friendster ($\mu = 0.60$)
Cross-sections
d
Friendster ($\mu = 0.50$)
Cross-sections
e
Friendster ($\mu = 0.40$)
Cross-sections
f
Friendster ($\mu = 0.30$)
Cross-sections
g
Friendster ($\mu = 0.20$)
Cross-sections
h
Friendster ($\mu = 0.10$)
Cross-sections
$\omega_{\mathrm{inter}}$
$\omega_{\mathrm{intra}}$
$\rho_\infty$
$\omega_{inter} = 0.1$
$\omega_{inter} = 0.3$
$\omega_{inter} = 0.5$

**Supplemental Fig. 12**. **Diffusion dynamics on the reshuffled Friendster social network with tunable community structure.** To investigate the impact of community structure, we employ a rewiring algorithm that preserves the degree distribution of the original network, while systematically varying the mixing parameter $\mu$ (the fraction of inter-community edges). This procedure randomizes the specific connections but maintains the statistical properties of the degree distribution. The figure displays phase diagrams (left columns) and cross-sections (right columns) of the final adoption density $\rho_\infty$ for various $\mu$. The cross-sections show $\rho_\infty$ as a function of the intra-community transmissibility $\omega_{\text{intra}}$ for fixed inter-community transmissibility $\omega_{\text{inter}} = 0.1$ (pink), $0.3$ (orange), and $0.5$ (green). The results show that the diffusion regimes and intervention patterns observed in the synthetic network models remain consistent across different levels of community mixing in the reshuffled empirical network.

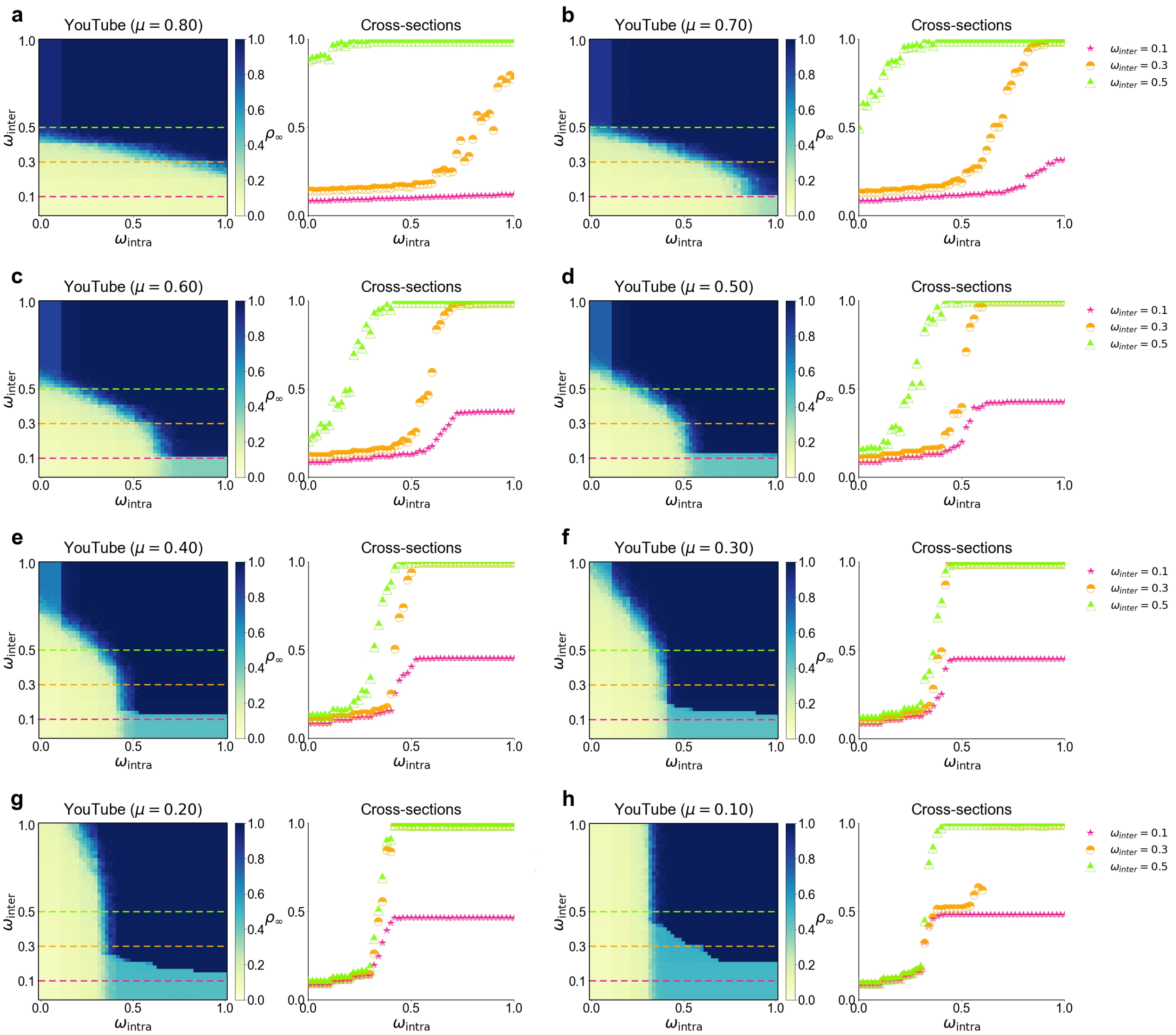
a
YouTube ($\mu = 0.80$)
b
YouTube ($\mu = 0.70$)
c
YouTube ($\mu = 0.60$)
d
YouTube ($\mu = 0.50$)
e
YouTube ($\mu = 0.40$)
f
YouTube ($\mu = 0.30$)
g
YouTube ($\mu = 0.20$)
h
YouTube ($\mu = 0.10$)
Cross-sections
$\omega_{\rm inter}$
$\omega_{\rm intra}$
$\rho_\infty$
$\omega_{inter} = 0.1$
$\omega_{inter} = 0.3$
$\omega_{inter} = 0.5$

**Supplemental Fig. 13**. **Diffusion dynamics on the reshuffled YouTube social network with tunable community structure.** The experimental setup, degree-preserving rewiring procedure, and plotting conventions are identical to those used for the Friendster network (Supplemental Fig. **12**). The results show that the diffusion regimes and intervention patterns remain consistent across different levels of community mixing in this empirical network.

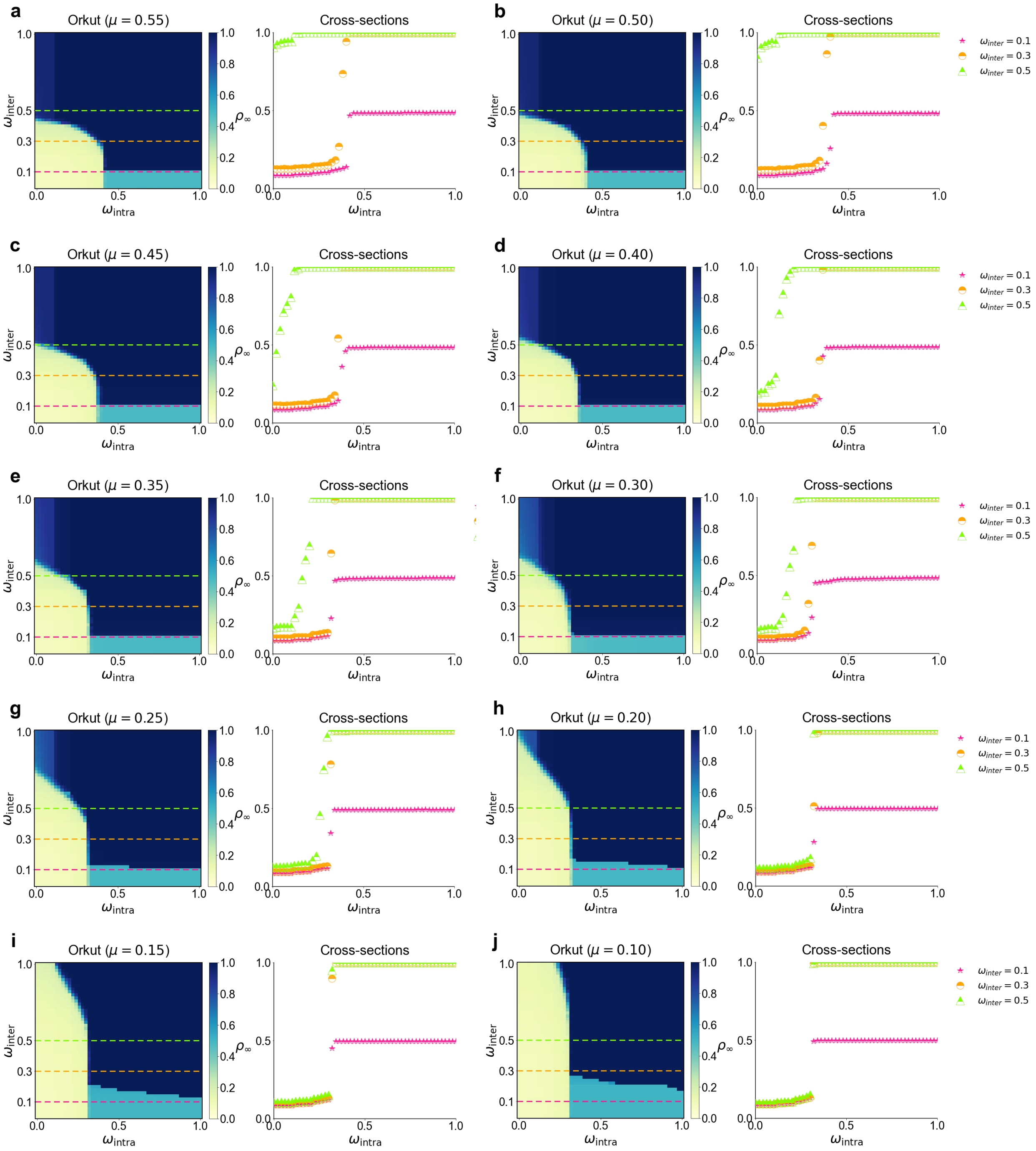
a
Orkut (μ = 0.55)
Cross-sections
b
Orkut (μ = 0.50)
Cross-sections
c
Orkut (μ = 0.45)
Cross-sections
d
Orkut (μ = 0.40)
Cross-sections
e
Orkut (μ = 0.35)
Cross-sections
f
Orkut (μ = 0.30)
Cross-sections
g
Orkut (μ = 0.25)
Cross-sections
h
Orkut (μ = 0.20)
Cross-sections
i
Orkut (μ = 0.15)
Cross-sections
j
Orkut (μ = 0.10)
Cross-sections
ω_inter
ω_intra
ρ_∞
ω_inter = 0.1
ω_inter = 0.3
ω_inter = 0.5

**Supplemental Fig. 14**. **Diffusion dynamics on the reshuffled Orkut social network with tunable community structure.** The experimental setup, degree-preserving rewiring procedure, and plotting conventions are identical to those used for the Friendster network (Supplemental Fig. **12**). The results show that the diffusion regimes and intervention patterns remain consistent across different levels of community mixing in the Orkut network.

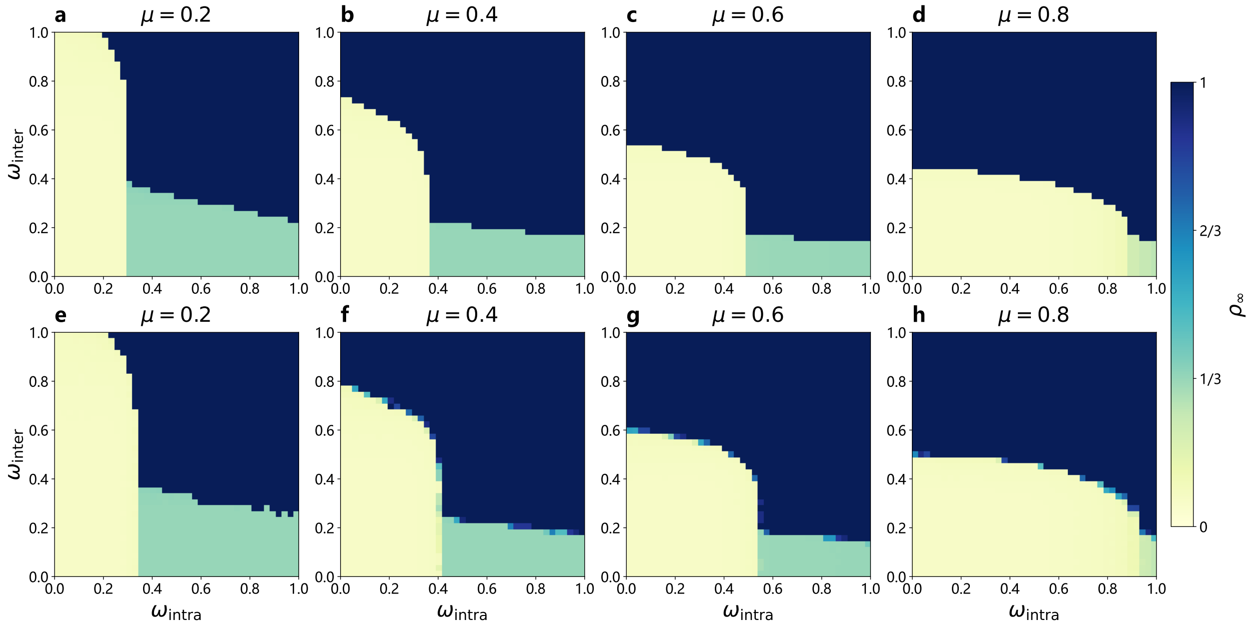


**Supplemental Fig. 15**. **Validation of the community structure–regulation coupling in three-module networks.** Phase diagrams of the final adoption density $\rho_\infty$ from the TL approximation are shown in panels (**a–d**), with corresponding Monte Carlo simulation results in (**e–h**) for mixing parameters $\mu = 0.2$, $0.4$, $0.6$, and $0.8$. We assume a symmetric configuration where the control parameters are uniform (identical $\omega_{\text{intra}}$ for all modules and identical $\omega_{\text{inter}}$ between all module pairs). The excellent agreement between theory and simulation confirms that the community structure–regulation coupling identified in two-module systems generalizes to multi-module topologies, demonstrating the robustness of the theoretical framework. Parameters: $\theta = 0.1$, $\rho_0 = 0.17$, $z = 20$, and $N = 20{,}000$ (simulations, averaged over 16 independent realizations).

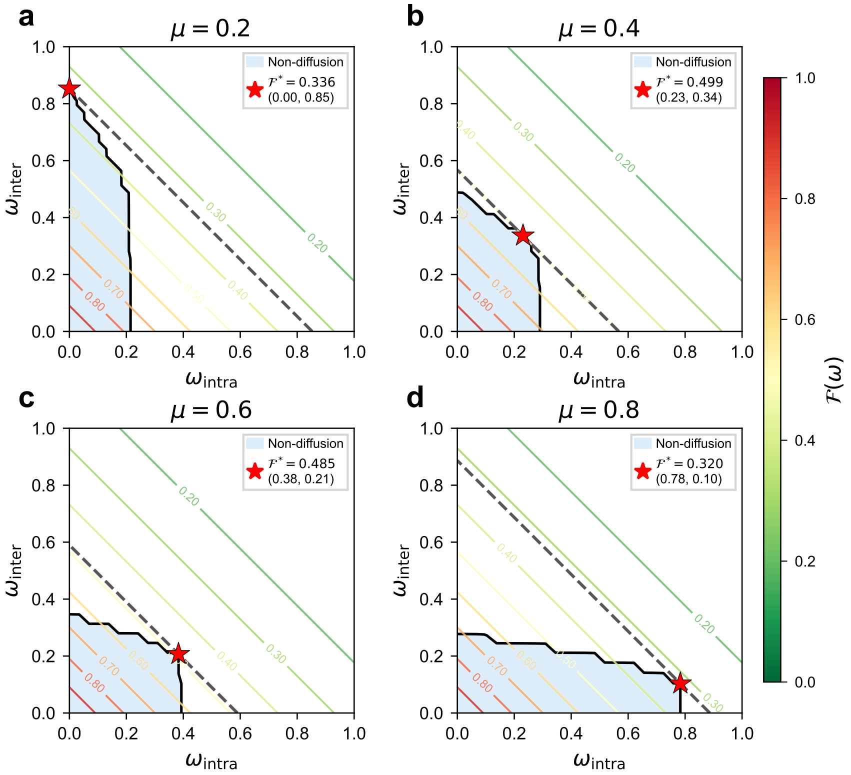


**Supplemental Fig. 16**. **Optimal intervention strategies across different community structures.** Each panel shows the $(\omega_{\text{intra}}, \omega_{\text{inter}})$ parameter space for a given mixing parameter $\mu$. The background color map indicates the cost function $\mathcal{F}(\omega)$, the light blue shaded region marks the non-diffusion phase, and the solid black line denotes the phase boundary. The parallel colored lines (decreasing in value from bottom-left to top-right) represent iso-cost contours of $\mathcal{F}$ with slope -1. The dashed gray line denote the specific contours tangent to the phase boundary, where the red star marks the optimal operating point that minimize $\mathcal{F}$, with its coordinates and corresponding $\mathcal{F}^*$ value indicated. (a) $\mu = 0.2$: the steep phase boundary places the optimum at $(\omega_{\text{intra}}, \omega_{\text{inter}}) = (0.00,\ 0.85)$, favoring strong intra-community control. (b) $\mu = 0.4$: the optimum shifts to (0.23, 0.34) as the boundary becomes less steep. (c) $\mu = 0.6$: the optimum moves to (0.38, 0.21), reflecting a transition toward inter-community control. (d) $\mu = 0.8$: the flatter boundary drives the optimum to (0.78, 0.10), favoring stronger inter-community regulation. This progression demonstrates a topology-driven shift in the optimal strategy, from prioritizing intra-community regulation in strongly modular networks to emphasizing inter-community control as modular structure weakens.
